# ‘I can feel myself coming out of the rut’: a brief intervention for supporting behaviour change is acceptable to patients with chronic musculoskeletal conditions

**DOI:** 10.1186/s12891-023-06336-7

**Published:** 2023-03-29

**Authors:** Amelia Parchment, Wendy Lawrence, Em Rahman, Nick Townsend, Elaine Wainwright, David Wainwright

**Affiliations:** 1 Department for Health, University of Bath, Bath, BA2 7AY England UK; 2 Aberdeen Centre for Arthritis and Musculoskeletal Health (Epidemiology Group), School of Medicine, Medical Sciences and Nutrition, University of Aberdeen, Aberdeen, AB25 2ZD UK; 3 Primary Care, Population Science and Medical Education, Faculty of Medicine, University of Southampton, Southampton, SO17 1BJ England UK; 4 Public Health Workforce Development, Southern House, Health Education England, Winchester, SO21 2RU England UK; 5 Centre for Exercise, Nutrition and Health Sciences, School for Policy Studies, University of Bristol, Bristol, BS8 1TZ England UK

**Keywords:** Musculoskeletal conditions, Chronic pain, Behaviour change, Self-management, Brief intervention, Physiotherapy

## Abstract

**Aim:**

To a) understand the perceptions and experiences of patients with musculoskeletal (MSK) conditions in relation to their physiotherapy care and their acceptability of ‘Making Every Contact Count Healthy Conversation Skills’ (MECC HCS) as a brief intervention within this care and, b) explore the mechanisms through which MECC HCS might facilitate behaviour change and enhance self-management in patients with MSK conditions.

**Methods:**

This study adopted an exploratory qualitative design, in which individual, semi-structured interviews with participants were conducted. Eight participants were interviewed. Five had been engaging with physiotherapists trained in and delivering MECC HCS within their routine physiotherapy appointments and three had been engaging with physiotherapists who had not received this training and were instead delivering usual care. MECC HCS is a person-centred approach to behaviour change that aims to empower individuals to take control of their health behaviours by building self-efficacy. The MECC HCS training programme helps healthcare professionals to develop skills in i) using ‘open discovery’ questions to explore context and allow patients to identify barriers and generate solutions; ii) listening more than giving information/ making suggestions; iii) reflecting on practice and iv) supporting Specific, Measurable, Action-oriented, Realistic, Timed, Evaluated, Reviewed (SMARTER) goal setting.

**Results:**

Those who had engaged with MECC HCS trained physiotherapists found their physiotherapy care highly acceptable and felt that their physiotherapist listened to them, tried to understand their context and world, and helped them plan for change. These individuals experienced increases in self-efficacy and motivation for self-managing their MSK conditions. A need for continued support following physiotherapy treatment was, however, emphasised for long-term self-management.

**Conclusions:**

MECC HCS is highly acceptable to patients with MSK conditions and pain and may successfully facilitate health-promoting behaviour change and enhance self-management. Providing opportunities to join support groups following physiotherapy treatment may promote long-term self-management and provide social and emotional benefits for individuals. The positive findings of this small qualitative study warrant further investigation on the differences in experiences and outcomes between patients engaging with MECC HCS physiotherapists and those receiving treatment as usual during routine physiotherapy care.

**Supplementary Information:**

The online version contains supplementary material available at 10.1186/s12891-023-06336-7.

## Introduction

Musculoskeletal (MSK) conditions are a growing public health concern and the leading cause of pain and disability in the UK [73]. Many are chronic in nature, lasting longer than three months [54, 67]. Their impact on the quality of life of sufferers [14, 23], and the associated economic burden faced by employers and health services [53, 60] mean that improving the management of these conditions is crucial. A shift from hierarchal, clinician-led physiotherapy care, focused on pathological ‘cure’, towards a more holistic, person-centred approach to care means that patients are now recognised as active, rather than passive, agents in the effective management of MSK conditions and pain [70]. Physiotherapists are thus encouraged to empower patients, using person-centred principles, to take responsibility for, and actively manage their own conditions [27]. This could involve supporting patients to take steps to change their behaviour and improve lifestyle factors such as sleep, physical activity and stress [26]. Such active self-management has been associated with reduced pain-related disability and use of healthcare services [6] and may be important for long-term management of MSK conditions and pain, when physiotherapy treatment has ended [27, 40].

Studies report that patients with MSK conditions and pain value listening and exploring individual context as important for facilitating behaviour change to self-manage [31]. Goal setting for increasing self-efficacy in self-management, and continued support from healthcare practitioners for offering encouragement and external accountability for change are also key facilitators [31, 64]. However, physiotherapists report difficulty integrating person-centred principles into practice [15], feel under-skilled in addressing psychosocial context relating to pain management [66] and do not always use effective techniques to empower patients to engage in productive behaviour change [28, 69]. It has been recommended that physiotherapists have more opportunities for training in patient-centred communication and learn how to integrate behaviour change techniques into practice in order to support health-promoting behaviour change and self-management in patients with MSK conditions and pain [24, 34].

Healthy Conversation Skills (HCS) is a practical, person-centred approach to behaviour change, based on empowerment and designed to support individuals to identify and take steps to change behaviours that are salient to them [3]. Underpinned by the Taxonomy of Behaviour Change Techniques [46], the HCS training programme helps healthcare professionals to develop skills in i) using ‘open discovery’ questions to explore context, and allow patients to identify barriers and generate solutions; ii) listening more than giving information/ making suggestions; iii) reflecting on practice and; iv) supporting Specific, Measurable, Action-oriented, Realistic, Timed, Evaluated, Reviewed (SMARTER) goal-setting. Delivery of HCS using these skills aims to empower patients to take control of their behaviours and build self-efficacy [2]. Higher self-efficacy is associated with healthier behaviours and increased self-management in those with chronic conditions [13, 42].

 Evidence suggests that HCS training increases the competence and confidence of practitioners in supporting behaviour change in patients and service users [4, 25, 36, 38]. Similarly, positive outcomes have been found for pregnant women receiving the intervention, who set more behaviour change goals and made more positive changes to their health behaviours than controls [1, 37]. High perceived acceptability of HCS has also been reported by staff delivering and service users receiving the brief intervention [30, 37]. Further research is, however, warranted to evaluate this brief intervention for a wider range of staff and, particularly, patient groups [51]. For example, despite HCS principles drawing from literature focusing on self-management in chronic conditions such as arthritis [3, 7], nothing has yet been done to evaluate the intervention for these patient groups.

HCS has recently been integrated as the main component of the Wessex model of ‘Making Every Contact Count’ (MECC); a government initiative to embed prevention, health promotion and self-management support into routine care [57]. Since MECC is now part of NHS standard contracts [49] and a priority in the UK government’s 5-year plan for improving MSK health [58], evaluating it in settings that support people with MSK conditions is important for understanding if, how and why this approach drives change and for informing enhanced future implementation [63]. A preliminary study investigating the uptake and perceptions of MECC HCS in physiotherapy services for the first time was promising [52] and showed that physiotherapists found the brief intervention highly acceptable, appropriate and feasible within their role for supporting patients with MSK conditions and pain. Moreover, a subsequent, qualitative study (submitted for publication) showed that physiotherapists found MECC HCS highly valuable in facilitating person-centred care for supporting behaviour change and self-management with these patients. Despite these promising findings, exploring and understanding the perspectives of patients is also important for evaluating the success of healthcare intervention implementation and remains a gap in the evidence-base for MECC HCS [61].

The aims of the present study were therefore to answer the following research questions:

1. What are the perceptions and experiences of patients with MSK conditions in relation to their physiotherapy care and their acceptability of MECC HCS as a brief intervention within this care?
2. What are the potential mechanisms through which MECC HCS might facilitate behaviour change and enhance self-management in patients with MSK conditions and pain?

## Methods

### Ethics

This study was approved by the Health Research Authority (HRA) and the Research Ethics Committee (REC) (reference 21/EE/0107) in addition to the University of Bath’s Research Ethics Approval Committee for Health (REACH) (reference EP 20/21 060). Informed written consent was sought via Online Surveys, prior to scheduled interviews. Verbal consent was additionally provided by participants on commencement of their interviews.

### Design

A qualitative research design was employed, within which individual, semi-structured interviews were conducted with participants. This design was chosen for the purpose of developing a rich, in-depth understanding of the unique experiences and perspectives of patients with chronic MSK conditions in relation to their physiotherapy care. Specifically, we hoped to explore their acceptability of MECC HCS within routine appointments, after training several MSK physiotherapy teams in this brief intervention as part of a wider project. Patient acceptability of healthcare interventions is an important indicator of implementation success and a prerequisite for further rollout [47]. This construct was therefore considered a highly relevant focus for the present study given that very little has been done to evaluate MECC for patients, despite its rollout being a priority in the NHS agenda [49]. A group receiving ‘treatment as usual’ during physiotherapy sessions was additionally included in this study design. Involving a group receiving treatment as usual, in addition to one receiving the intervention of focus, in qualitative healthcare research has been recommended for assessing the potential impact of an intervention and its key ingredients for driving change [41].

This study was underpinned by critical realism, which recognises that whilst an objective, universal reality might exist, this cannot be accessed by the researcher. Rather, only subjective, situated perceptions and interpretations of reality can be studied and shape what is known about the world [44]. This subjectivity relates not only to the perspectives of participants involved in research but also the researchers who construct the findings [56]. An understanding of both context within which experiences occur, and the influence of the researcher is therefore important. This critical realist approach informed the use of reflexive thematic analysis to provide an interpretation of the data.

### Participants

#### ‘Intervention’ participants

Between October 2021 and December 2021 physiotherapists in several UK NHS trusts received MECC HCS training as part of a wider project (submitted for publication) which aimed to evaluate this brief intervention in physiotherapy services. The lead researcher (AP) liaised with these physiotherapists to identify patient participants for the present study, with whom they would deliver MECC HCS during scheduled appointments. Eligible patients had any MSK condition and/or MSK pain lasting over three months (therefore defined as ‘chronic’ [50]) and were receiving NHS physiotherapy treatment over several sessions. Potential participants were informed of this study when attending their first physiotherapy session. Those who expressed interest in the study were given the relevant information by their physiotherapist to take home and consider. This information gave details of the study and directed those interested in participating to contact the lead researcher. Potential participants were also given the option for the lead researcher to contact them. In this case, they consented to be contacted via their physiotherapist/ clinical team. Participants were given the opportunity to ask any questions and further discuss their involvement in the study with the lead researcher, before a convenient interview date and time was arranged. These interviews took place at the end of their physiotherapy treatment period.

#### ‘Treatment as usual’ (TAU) participants

A group receiving usual care was included in this study, as recommended for qualitative studies aiming to explore the impact and key ingredients of healthcare interventions for patients [41]. Participants in this group met the same eligibility criteria as those in the ‘intervention’ group but instead received TAU from their physiotherapists over a number of sessions. These physiotherapists had received no training in MECC HCS and were delivering usual care. Potential TAU patient participants were identified through physiotherapy services with which the lead researcher was liaising as part of the wider project mentioned above. Similar to ‘intervention’ participants, they were directed to contact the lead researcher if interested in taking part in the study and an interview was scheduled for the end of their physiotherapy treatment period.

### Data collection and analysis

Interviews were conducted by the lead researcher between April and July 2022 and lasted between 22 and 68 min, with an average length of 38 min. Most interviews took place via Microsoft Teams (6/8, 75%), however, due to technical issues, two were conducted via telephone (2/8, 25%). With permission from participants, all interviews were audio-recorded for transcribing purposes. Following a semi-structured interview guide (Supplementary File 1), the lead researcher asked open-ended, exploratory questions to allow participants to lead the discussion and reflect on i) their experiences of their physiotherapy care; ii) interactions with their physiotherapists; iii) what was discussed during these interactions; iv) how their own values, needs, goals, and concerns were addressed; v) how they were supported to take steps to change behaviours and/ or self-manage pain; vi) what they thought about brief, supportive behaviour change interventions being introduced to routine physiotherapy care, and; vii) what could be done to improve physiotherapy care for those living with chronic MSK conditions and pain.

Interviews were transcribed verbatim and anonymised at the point of transcription. The lead researcher then employed reflexive thematic analysis [8], using both an inductive and deductive approach to explore patterns of meaning within the data. The analysis was situated within a critical realist perspective, holding that reality can only be accessed and studied through the ‘filter of human experience and interpretation’ ([18], p.183). This complements the focus of reflexivity within our analytical approach, whereby the lead researcher frequently reflected critically upon her own involvement/subjectivity and interpretation in the qualitative analysis through the use of reflexive journaling and engaging regularly in discussion with the other authors (‘critical friends’) regarding the development of themes. The purpose of this was to enhance critical reflection of her situated, subjective interpretation of reality and challenge her context-dependent construction of knowledge, rather than to achieve an objective consensus regarding theme development. The lead researcher was, for example, a 26-year-old PhD researcher who was early in her research career and had been developing skills in reflexive thematic analysis for the duration of her studies. She had not experienced chronic pain herself but was personally invested in this topic due to having relatives and friends who did experience chronic pain. The use of reflexive journaling and engaging with ‘critical friends’ was considered an appropriate approach to reflexive thematic analysis, given that subjectivity is an important tool for this particular qualitative method [8].

Following Braun and Clarke’s [8] six stages of reflexive thematic analysis, the lead researcher began by immersing herself in the data, reading and re-reading all interview transcripts. Next, meaningful patterns in the data were explored and initial codes developed. This initial stage was done after mixing the order of transcripts and coding the whole dataset rather than looking at whether or not participants had engaged with physiotherapists trained in MECC HCS. This was to reduce researcher bias of expecting those who had been exposed to the brief intervention to have different perceptions and experiences of their physiotherapy care, distinct from those who had not. However, since the lead researcher had conducted all interviews with participants, this bias could not be completely eliminated. Analysis of the whole dataset in the initial coding stage preceded the comparing and contrasting of data, between groups, and development of broader themes. Code and theme development began inductively (data driven), and this was felt to be a beneficial start-point for exploring respondent/data-based meaning, before deductive analysis ensured that developed themes were meaningful in relation to the posed research questions. Themes were developed in an iterative process, whereby they were reviewed and refined until it was felt that they accurately represented the meaning in the data. Themes were then defined, named, and exemplified before the analytic account was written.

## Results

### Demographics

Eight patient participants were interviewed. They were aged between 24 and 74 (M = 56.5) and mostly female (6/8, 75%). All were receiving NHS physiotherapy care in the South-West (7/8, 87%) or the South-East (1/8, 13%) due to chronic MSK conditions and/or pain lasting over three months. Five (63%) were attending appointments with physiotherapists who had received MECC HCS training. Three (37%) were attending appointments with physiotherapists who had not been trained in MECC HCS and were instead delivering ‘treatment as usual’. Participant characteristics are described in Table 1.

**Table 1:** Participant characteristics

Participant	Group	Gender	Age (years)	Ethnicity	Diagnosis	Employment status	Location
1	Intervention	Female	56	Mixed White and Asian	Fibromyalgia	Unable to work due to sickness or disability	South-West
2	Intervention	Male	24	White British	Knee pain	In paid employment or self-employed	South-West
3	Intervention	Female	74	White British	Fibromyalgia, Peritoneal Fibrosis, Scoliosis, Osteoarthritis	Retired	South-West
4	Intervention	Female	54	White British	Knee pain	Unable to work due to sickness or disability	South-West
5	Intervention	Female	62	White British	Hip pain	In paid employment or self-employed	South-East
6	TAU	Female	57	White British	Osteoarthritis	Retired	South-West
7	TAU	Male	65	White British	Osteoarthritis	In paid employment or self-employed	South-West
8	TAU	Female	60	Asian British	Knee pain	In paid employment or self-employed	South-West

### Themes

Three main themes were developed during reflexive thematic analysis [8], which encapsulate the perspectives of patients with MSK conditions in relation to their experience of physiotherapy care. Themes comprised: Theme 1) ‘She sat, and she listened’: exploring context facilitates person-centred care, Theme 2) Motivation for moving forward: the value of setting goals and 3) ‘Now I’m back out in the wilderness’: the need for further support. Themes are presented in turn below, with quotations provided as exemplars of our interpretation. Pseudonyms are used to protect participant identities.

### Theme 1: ‘She sat, and she listened’: exploring context facilitates person-centred care

All participants emphasised the importance of treating patients as individual people for productive physiotherapy care. This involved physiotherapists adopting a holistic approach, through which they understood patients not only as the isolated problem they were presenting with, but instead whole beings with different psychological, social, and physical needs.

‘If you just think of Pinocchio, you’ve got to understand all of his strings to understand him. You can’t just do one string cos’ they’re all interdependent.’ (Participant 8).

To enable this approach, participants highlighted the value of listening. Those who engaged with MECC HCS- trained physiotherapists consistently felt listened to, allowing for their physiotherapist to gain a deeper understanding of their individual context and how this might impact their experience and management of pain.

‘She really did listen… she was coming from a position of, you know, trying to understand, and she really took on board, or listened to, you know, how I manage various things. Obviously, at that time, I was in a house with stairs, so that was a really big… you know, your life becomes consumed by a set of stairs.’ (Participant 1).

Through gaining this understanding and developing a holistic picture of the patient and their unique world, physiotherapists were able to support planning for change in a person-centred way. Physiotherapists used ‘open discovery’ questions to facilitate this, allowing participants to explore and reflect on their own context and experiences, identify steps to productive change and solutions to problems:

‘She made a point of finding out my lifestyle… how active I was… what kind of job I did… what other activities I do… I mentioned I did start walking during COVID and I went with my friend and that made me go because she was going through breast cancer, so I was trying to get out for her to help with her mental health, but she walked really quickly and I was finding that by walking… that what was what seemed to aggravate my hip. You know, when you’re walking extra fast and erm, we [physiotherapist and I] decided not to give up walking but to start again by first walking at a slower pace…’ (Participant 5).

As reflected by the use of the word *‘we’* in the quotation above, several participants seemed to engage in a process of collaborative, shared decision-making with their physiotherapist*, ‘working together’* (Participant 1) to find ways to improve physical and/or mental health and wellbeing and enhance pain self-management. Participant 3 described how she felt empowered during and after this process to improve dietary quality in order to lose weight. This empowerment was achieved through being actively involved in identifying ways in which she could improve health behaviours and feeling supported in her journey towards achieving this:

‘She never told me what to do or what she thinks I should do; she guides me along and listens to what I have to say… I feel she’s made me a bit stronger.’ (Participant 3).

In contrast, despite similarly highlighting their perceived value of a patient-centred, collaborative approach to supporting behaviour change, participants who had engaged with TAU physiotherapists did not report this within their consultations. Rather, these participants discussed how their physiotherapists engaged less in listening, more in information delivery and adopted a ‘one size fits all’ approach to care:

‘I could have read what I spoke to the physio about on the internet… he’d give me information, he’d give me sheets… Just take a bit more care. You know, I’m an individual. I know he sees lots of people for lots of different physio things but this is me, this is my knee, this is how it’s happening with me. Please listen and adapt what you do with a hundred other people to me, You know, don’t just blanket everything.’ (Participant 6).

This approach was problematic when thinking about taking steps to improving health and/or enhancing self-management, since participants emphasised the importance of recognising individual needs, goals, values, and challenges when supporting change. Participant 8, for example, discussed the battle she had faced with managing her weight, despite knowing the impact it has on her chronic knee pain. The telling/ suggesting approach to facilitating behaviour change adopted by her physiotherapist was considered unhelpful and unproductive since it did not acknowledge her personal barriers to change or help her to identify how to overcome these barriers:

‘The way it was dealt with was via link to a website that was all about the issues to do with joint care.. it’s not much better than someone telling you what to do because you’re not gonna do it until you’ve maybe dismantled the barriers as to why you’re not doing it. If I’m overweight, I’m overweight because I haven’t worked out how not to be overweight so you telling me to do the right things, there are barriers there.’ (Participant 8).

### Theme 2: Motivation for moving forward: the value of setting goals

Participants that engaged with MECC HCS trained physiotherapists discussed in depth how they were supported to set personal goals that were followed up in consecutive physiotherapy appointments. Many emphasised the value of this goal setting for increasing motivation, particularly when taking first steps to change. Participant 3 described how she had previously become consumed by her chronic pain, unable to find strength or motivation to do anything she enjoyed. Increasing motivation through setting small, personal goals with her physiotherapist had a positive impact on both her physical and mental health and wellbeing:

‘Like she's been, ‘what goals would you like to reach?’ And I'd say, well, I'd like to go out walking more and sort of perhaps walk… walk to my son’s house and back or just round the blocks for like a 20 min walk…So I do set those sort of goals with her. Because she always asked me, the next time I see her, how I've got on with doing these goals that we've discussed…I think mentally I was going downhill. I would just cry at the… you know, anything. I do find [goal-setting] useful, because it makes you do it… you know, it's pulling me out of this rut that I've got myself in to…it makes me… it makes me motivated, to start with. And I can feel myself coming out of the rut.’ (Participant 3).

Through developing mastery experience, goal setting also increased participants’ self-efficacy for engaging in health-promoting behaviours, such as physical activity. This, for some, reduced fear- avoidance, which had previously resulted in disengagement from hobbies and/ or becoming sedentary due to fear of increased pain or further injury. Goal setting in physiotherapy care thus seemed to additionally facilitate longer-term, active self-management of pain:

‘And also running as well is something that I'm doing recently. Not like lots of running but just sort of… just again to make me feel more confident. So, I guess it's also having a big impact on my mental health probably because like before, as I was saying, like I was not very confident really and now I definitely feel like within six months or so I feel a lot more confident doing like physical things… I guess I always felt like I might injure my knee again, so I always played it very safe I guess… ever since I had the knee injuries, I really relaxed it quite a lot. For me personally, it [goal setting] keeps me motivated and makes sure that I'm on the right track to my long-term goals, which is obviously making sure that I'm getting like less pain and that sort of thing.’ (Participant 2).

When improving health behaviours was not the priority, goal setting for enhancing self-management instead focused on preventing cycles of ‘*boom and bust’* (Participant 1). Participants described themselves as overexerting themselves on days on which they were in less pain, leading to severe pain flare-ups and fatigue in the following days. This led to a cycle of fewer ‘good’ pain days and more prolonged, debilitating, "bad"  pain days. Participant 1 described how setting specific, action-oriented goals during physiotherapy sessions helped her to avoid ‘boom and bust’ and manage her chronic pain more effectively:

‘What I tend to do is, because I’m better in the mornings… because of the painkillers and then taking the dogs out, being outside sort of makes you feel better. So, I would generally just keep working and doing things, because the minute I sit down, everything starts- the pain, the this, the that. So yeah, we did goal setting in order to try and manage things a little better and recognise things…setting an alarm, so that an alarm goes at 2 o’clock and that tell you, right, now I need to start winding down…’ (Participant 1).

Finally, intervention participants reflected on how they felt there was no quick fix or cure for their chronic pain, which was unpredictable, ever-fluctuating and often-debilitating in nature. However, having personal goals to focus on, follow up, build upon and achieve, provided hope and optimism for the future, *‘even down to the silly things like doing [participant’s] own shopping’* (Participant 4):

‘I just want to carry on and just build up my strength and just go from strength to strength until I can hopefully- well, I know I won’t ever be the same person I was again, but get back to… you know, get back into the, well, the big, wide world I suppose.’ (Participant 3).

Again, there were differences between the experiences of the above participants and those who attended appointments with TAU physiotherapists. Whilst all intervention participants discussed setting goals with their physiotherapists and being supported in planning for change, those in the TAU group either set no goals at all or focused only on targeting biomechanical factors for short-term recovery:

‘Yeah, there were no goals set or anything… I suppose just the physio itself was the only goal really, you know, just keep doing the physio, keep working at it.’ (Participant 6).

### Theme 3: ‘Now I’m back out in the wilderness’: the need for further support

The third and final theme encapsulates how follow up support from physiotherapists was perceived as integral to participants for enabling long-term, productive behaviour change and management of pain. However, both intervention and TAU participants reported feeling forgotten about by the health service following their final physiotherapy appointments. A sudden end to support conflicted with the long-term nature of symptoms experienced by participants and the social and psychological impact of living with a chronic condition. Participant 1, for example, discussed feeling ‘dropped’ by her physiotherapy service, ‘alone’ again in navigating life with chronic pain and trying to self-manage:

‘…Three months later [after physiotherapy starts] it’s like, well, you know, thanks and goodbye. Now what? Okay, I’ve got all these tools and I’m trying to implement them, but there’s no follow-up six, nine, twelve months down the line to say, yeah, you are on the right track… it’s very difficult… it is a chronic condition, so you’re not going to get better from this.’ (Participant 1).

For some, complete loss of contact with physiotherapists following the treatment period meant that progress made in managing pain was reversed, symptoms intensified, and, in turn, participants’ need for further treatment increased:

‘…all of a sudden it just all went backwards and downhill and I was in a lot of pain and I couldn't move my knee. I couldn't get an appointment with the physio, so again I had to pay and go to the one at the hospital…when I needed to see him, there wasn’t an appointment available.’ (Participant 6).

Participants thus advocated for occasional follow up check-ins from their physiotherapists upon completion of allocated appointments to a) ensure progress in pain self-management and achieving long term goals, as discussed by Participant 2, and b) enable a sustained, therapeutic relationship between themselves and their physiotherapist, within which they had formed a sense of understanding and trust. This is reflected by Participant 3, who emphasised the chronicity of her condition and the need for continuity of care.

‘…it just helps a lot seeing someone about it and then getting feedback and then seeing how I'm doing…, I think the biggest thing really for me is just seeing someone.’ (Participant 2).

‘…just for her to carry on and support me, I suppose. I just feel she supports me so much that to carry on, so, you know, I’ve still got somewhere to go and someone to see that knows about me now’ (Participant 3).

The remit of the physiotherapists’ role, capacity limitations within physiotherapy services and costs associated with such extended support were, however, recognised. Some participants thus had alternative suggestions for how they could be supported with productive behaviour change and managing their condition following physiotherapy discharge. Emphasising that *‘prevention is better than cure’* (Participant 5), the potential value of opportunities such as support or exercise classes for people with similar MSK conditions, and health coaching provisions for those living with chronic pain were discussed. These opportunities were believed to have promise for offering a sense of belonging for those living with chronic MSK conditions, an increased understanding of these conditions and their symptoms, and a sustained element of accountability for self-managing them:

‘It would’ve been nice… I don’t know, if there was a class…not necessarily run by her [the physiotherapist], but if they could run like a six-week course or something for people with that condition… I’m not expecting anything for free but maybe it could help you with that particular condition. If they did pursue that, you know, people might be willing to pay a small fee and I think it would help people a lot to be honest, and the NHS in the long run.’ (Participant 5).

## Discussion

The aims of this study were to explore and understand i) the acceptability of implementing MECC HCS in physiotherapy care for patients with MSK conditions and pain, and; ii) the potential mechanisms through which MECC HCS facilitates behaviour change and self-management in these patients.

Firstly, participants who engaged with MECC HCS trained physiotherapists consistently felt listened to (HCS Skill 2) during consultations. They valued this highly, and believed it enabled their physiotherapists to develop a holistic understanding of them as individuals in relation to their experience and management of pain. This finding aligns with studies that have highlighted listening as a key communication skill, advocated for in healthcare settings by patients with MSK conditions and pain [16, 19, 31]. These patients report feeling at ease in clinical interactions when they are listened to and able to discuss their worries and concerns [16]. Moreover, they believe their individual circumstances can be better understood [31]. Finally, it is felt that through listening, a mutual respect between themselves and the healthcare professional can be established, enhancing their therapeutic relationship [19]. These examples suggest that listening may be a key HCS for facilitating high-quality person-centred care in MSK consultations [48]. Focusing on individual preferences, needs, beliefs, and experiences [45], person-centred care improves patient wellbeing and health outcomes [72] and is highly important to those living with MSK conditions and pain [10, 33].

The MECC HCS approach adopted by physiotherapists additionally seemed to facilitate behaviour change in patients. Participants reported being asked ‘how’ and ‘what’ questions (HCS Skill 1; ‘open discovery’ questions) to explore their own issues, identify steps to change that were important to them, and generate solutions to personal barriers to change. Engaging in a process of shared decision making when taking steps to change made patients feel they were actively involved and taking a lead role in their journey towards self-management but supported along the way. This led to a sense of empowerment. In contrast, those engaging with TAU physiotherapists reported receiving a telling/suggesting, ‘one size fits all’ approach to consultations, consisting mostly of information delivery. This approach was found to be lacking person-centredness and unhelpful for supporting productive behaviour change. MECC HCS is an approach to behaviour change that recognises that information alone is insufficient to change behaviour and that people must be motivated and able to change [32, 43]. Problem-solving is one behaviour change technique employed by healthcare professionals in the delivery of MECC HCS, enabling patients to identify their own barriers to change and solutions to these barriers [46]. Both problem-solving [9], and collaborative, shared decision making between patients and their physiotherapists [26] can enhance self-efficacy for changing behaviours. This self-efficacy, or one’s belief in their ability to do something [2], is associated with patient self-management [13, 42].

Secondly, participants that engaged with MECC HCS trained physiotherapists reported more long-term goal setting than TAU participants, who did not set goals or focused only on short-term symptom ‘cure’. Although not explicitly discussed by participants, those in the intervention group reflected on goal-setting that was specific, measureable, action-oriented, realistic, timed, evaluated and reviewed (SMARTER; HCS Skill 3) in nature. These goals were important and relevant to participants and supported longer-term management of conditions. Many discussed how they felt more motivated to self-manage when setting these personal goals during appointments, supporting self-determination theory [11]. This theory posits that motivation to change behaviour is increased when goals are important to, or meet the intrinsic needs, of the individual. Long-term change is then more likely [11].

Setting goals and regularly reviewing them with physiotherapists seemed to additionally increase self-efficacy for self-management and reduce fear avoidance in patients. These findings support literature demonstrating the value of patient-led goal setting, based on the preferences and agenda of the individual (and not those of the healthcare professional's or treatment recommendations), for improving quality of life, pain intensity, self-efficacy and fear avoidance in patients with chronic pain [20, 21]. Fear avoidance, or the avoidance of movement and/or activity due to fear of pain or further injury, is one psychosocial factor that is used to explain the development of chronic pain [22]. It is significantly associated with higher pain intensity [35], long-term sick leave [68], disability and depression [62]. Higher pain self-efficacy, or one’s ability to manage pain and engage in routine activities, is associated with lower fear-avoidance [55]. Through setting personal, valued goals, it is possible that participants developed mastery experience for changing behaviours and enhancing self-management [21]. Developing such mastery experience is likely to have enhanced self-efficacy [2] which, in turn, may have reduced fear-avoidance. Future MECC HCS evaluation should consider exploring the association between goal setting, self-efficacy and fear-avoidance further, and measuring factors such as pain intensity, sick leave, disability and depression as long-term outcomes. Conducting such evaluation would additionally help to address the currently large gap in the literature regarding the impact of MECC HCS on health and work outcomes in patients with MSK conditions and pain.

Finally, goal setting during physiotherapy appointments seemed to enhance hope for the future and mental wellbeing in participants. This supports other literature highlighting goal engagement for improving mental well-being in individuals with chronic pain [29] and goal setting for encouraging hope in patients with long term conditions [71]. The worrying association between chronic MSK pain and depression and anxiety is well documented [39, 74], whilst hope and optimism have been found to positively correlate with pain outcomes and quality of life [59]. Adopting HCS and supporting goal setting in physiotherapy care may provide an important opportunity to promote optimism, hope and mental health in patients with chronic MSK conditions and pain and enhance long-term self-management.

The third and final theme encompassed the concerns of participants in both the intervention and TAU groups in relation to life beyond physiotherapy treatment. Many discussed feeling like they had been abandoned by physiotherapy services following their last session and emphasised the need for ongoing support from physiotherapists for long term management of conditions, continuity of care and external accountability in relation to improving health behaviours. This aligns with other qualitative research involving those with chronic MSK pain. A recent systematic review, for example, discussed the facilitators to self-management in these patients [64]. These facilitators included ongoing encouragement or reassurance from the professional, the healthcare professional’s understanding of the patient’s specific needs and external accountability to start and continue self-management, offered by the professional. A key conclusion drawn from the review was the importance of continued support from healthcare professionals in facilitating self-management in patients with chronic pain.

The rapidly increasing number of individuals with MSK complaints does, however, mean that physiotherapy service demands are growing, and service capacities are becoming more limited [5]. The consequence of this is long wait lists for patients accessing physiotherapy services [12], and these waitlists have been shown to have a detrimental effect on health outcomes for those with MSK conditions [12]. These service limitations, and the resultant necessity of patient discharge was recognised by participants. Some therefore provided suggestions as to how they could alternatively be supported following the physiotherapy treatment to manage their chronic pain. A key suggestion was support and exercise groups involving other individuals with chronic MSK conditions. These types of groups enable people with similar problems to learn from each other whilst sharing experiences and concerns. Studies involving those with chronic pain have demonstrated the positive impacts of support groups, including enhanced functional ability and activity and decreased recourse to health professionals [65]. They have additionally been found to enhance sense of belonging and reduce isolation in these individuals [17]. Making support groups more accessible following physiotherapy treatment may be a cost-effective, valuable way of enhancing self-management, reducing health-service use and providing emotional and social benefits to those with chronic MSK conditions and pain [17].

### Limitations

The main limitation of this study was the possibility of recruitment bias. Most patients eligible for this study were identified by their physiotherapists. Physiotherapists may have therefore unintentionally or intentionally recommended patients to this study based on whether they expected them to engage well with the brief intervention. Secondly, those who expressed interest in the study may have participated due to the influence of social desirability and because their physiotherapist had encouraged them to. Participants were also recruited mostly from one region in England and may not represent the experiences and perspectives of individuals in different areas, such as those with lower or higher levels of deprivation and those from minority ethnic groups. Future research should work hard to engage individuals living in different levels of deprivation and from a range of underrepresented groups in order to enhance the likelihood of study findings and inferences being translated into the real-world. Since the prevalence of MSK conditions and pain is higher among some ethnic minorities and those living deprivation, engaging individuals from these groups and areas in MECC HCS evaluation could provide important contributions to supporting the long-term reduction of MSK health inequalities. Finally, although this data was collected following the COVID-19 pandemic, NHS services and trusts were still facing unprecedented capacity issues. This meant that physiotherapists’ time for supporting this research, and helping to identify eligible patients was limited, as was that of the services in which they were working. The limitations of the lead researcher as a PhD student in accessing patients directly, her reliance on physiotherapists for this access, and the COVID- impacted time limits associated with this study were all significant challenges to recruitment and lead to a limited sample size, particularly in the TAU group. However, this was an exploratory study that aimed to explore potential differences in findings between patients engaging with MECC HCS trained physiotherapists vs TAU physiotherapists and not to make generalisable truth claims in the same way a controlled, quantitative study would. Positive findings of this small, qualitative study could inform further investigation on the acceptability and impact of MECC HCS of patients with MSK conditions and pain using a more controlled design.

## Conclusion

This qualitative study illustrates that MECC HCS is a brief intervention that is acceptable to patients with chronic MSK conditions and pain within physiotherapy care. Our findings demonstrate that listening and asking ‘open discovery’ questions as key healthy conversation skills facilitate a person-centred approach to care and can empower patients to identify and take steps to productive behaviour change. Moreover, patient-led SMARTER goal setting as a healthy conversation skill increases motivation and self-efficacy for self-management. It also usefully targets psychosocial factors relating to the experience and management of pain, including mental wellbeing, fear avoidance and optimism. Finally, we suggest that more should be done to provide opportunities for those living with MSK conditions and pain to join support groups. These groups may be a cost-effective way to facilitate long-term self-management, reduce burden on physiotherapy services, and support social connectedness for those living with MSK conditions and pain.

## Supplementary Information

**Additional fie 1: Supplementary file 1.** Semi-structured interview guide.

## Data Availability

Materials (i.e., interview guide) are available from the corresponding author upon request.
