# Supplementary material for: ‘I can feel myself coming out of the rut’: a brief intervention for supporting behaviour change is acceptable to patients with chronic musculoskeletal conditions
Source: BMC Musculoskelet Disord. 2023 Mar 29;24:241. doi: 10.1186/s12891-023-06336-7 (PMC10050805; doi:10.1186/s12891-023-06336-7)
Supplement: Supplementary file 1 — Additional fie 1: Supplementary file 1. Semi-structured interview guide. [file 12891_2023_6336_MOESM1_ESM.docx]

**Supplementary File 1:** Semi-structured interview guide

**Welcome**

Hello and thank you for taking the time to talk to me today. My name is [lead researcher’s name], and I am a PhD student at the University of Bath, researching a brief intervention for people with musculoskeletal conditions and related pain. Today I am hoping to have a discussion about your current physiotherapy care, and typical appointments with your physiotherapists. I would also like to discuss your thoughts on health, wellbeing, and lifestyle being discussed during routine appointments, as well as how useful and acceptable this is/would be for you. You have been invited to take part because you have a musculoskeletal condition, related pain and are receiving physiotherapy care within an NHS trust. As stated in the participant information pack, I would like to record this interview as I don’t want to miss any of your comments. People often say very helpful things in these discussions and I can't write fast enough to get them all down. This recording will, however, be deleted as soon as I have transcribed and have checked the transcription. I’d like to remind you that all information will be kept strictly confidential, and that only the research team will have access to the information provided. All of your personal information will be protected and any directly identifying information, such as names, will be safeguarded and maintained under controlled conditions, with data being anonymised and any direct quotes from you in reports or publications will be de-identified. As per University of Bath guidelines I must highlight that, in the unlikely event that there is a safeguarding issue, and I feel that any of yourself or others are at risk of harm, this will be referred to the appropriate agencies- my supervisory team in the first instance. Although the interview is not designed to be in any way upsetting or too tiring, we can stop at any time you like for a break, and we can stop altogether at any point if need be. Do you have any questions?

Finally, I’d like to thank you so much for your interest and participation in this important research. All of your contributions are hugely appreciated, and I look forward to this discussion. Let’s begin.

**Questions and prompts**

1. What are your thoughts on the physiotherapy care you receive at the moment?

How have you found your current/recent physiotherapy sessions, particularly in supporting you to improve your health/well-being/symptoms (whichever fits best)?

1. How helpful are your conversations with your physiotherapist during your appointments?

How have the conversations you’ve had with your physiotherapist helped you to manage/improve your symptoms/condition?

1. How productive and supportive are these conversations, in your opinion?

How supported have you felt by your physiotherapist, particularly in relation to valuing your views and ideas?

1. When attending physiotherapy appointments, what lifestyle behaviours have you discussed, if any? i.e., physical activity, smoking, healthy eating, mental health, alcohol, weight
2. What happened during these conversations, if you’re happy to share?

How did the physiotherapist introduce any of these topics and how did the conversation go?

1. How much did you feel your physiotherapist listened to you during these conversations?
2. How much did you feel they found out about you and your world?

How much did you feel they found out about you, any concerns you had, your living/working situation and any thoughts you had about the best care for you?

1. What, if anything, did they do to help you take first steps to change or help you plan for change?

What did they do to help you plan first steps towards doing things differently or making a change in order to improve your symptoms/condition/health?

1. What would make it easier to have or maintain a healthy lifestyle and mental wellbeing?

(if needed for the interviewer: i.e. exercise classes in the community, wellbeing groups in the community, additional time with a specific healthcare provider)

1. What, if anything, would you like to discuss more with your physiotherapist, with regard to your health and wellbeing?
2. How could appointments, particularly conversations with your physiotherapists during your routine care, be improved to best suit your needs?
3. How would you feel about brief, supportive, behaviour change interventions, delivered by your physiotherapist, being part of routine care? (This might involve simply discussing lifestyle factors and how they can affect your MSK health to setting behaviour change goals and following these up over a number of weeks during your routine physiotherapy appointments)
4. What final comments, if any, do you have regarding your current physiotherapy care, the conversations you have with your physio, what you would improve or anything else?
5. Is there anything else we have not covered that you would like to say about this research topic or the study?
